# Optimizing exoskeleton assistance to improve walking speed and energy economy for older adults

**DOI:** 10.1186/s12984-023-01287-5

**Published:** 2024-01-02

**Authors:** Ava Lakmazaheri, Seungmoon Song, Brian B. Vuong, Blake Biskner, Deborah M. Kado, Steven H. Collins

**Affiliations:** 1 Department of Mechanical Engineering, Stanford University, Stanford, CA USA; 2 Department of Mechanical and Industrial Engineering, Northeastern University, Boston, MA USA; 3 Geriatrics Research Education and Clinical Center, Veterans Affairs, Palo Alto, CA USA; 4 Department of Medicine, Stanford University, Stanford, CA USA

**Keywords:** Exoskeletons, Aging, Gait speed, Energy cost, Human-in-the-loop optimization, Motor learning

## Abstract

**Background:**

Walking speed and energy economy tend to decline with age. Lower-limb exoskeletons have demonstrated potential to improve either measure, but primarily in studies conducted on healthy younger adults. Promising techniques like optimization of exoskeleton assistance have yet to be tested with older populations, while speed and energy consumption have yet to be simultaneously optimized for any population.

**Methods:**

We investigated the effectiveness of human-in-the-loop optimization of ankle exoskeletons with older adults. Ten healthy adults > 65 years of age (5 females; mean age: 72 ± 3 yrs) participated in approximately 240 min of training and optimization with tethered ankle exoskeletons on a self-paced treadmill. Multi-objective human-in-the-loop optimization was used to identify assistive ankle plantarflexion torque patterns that simultaneously improved self-selected walking speed and metabolic rate. The effects of optimized exoskeleton assistance were evaluated in separate trials.

**Results:**

Optimized exoskeleton assistance improved walking performance for older adults. Both objectives were simultaneously improved; self-selected walking speed increased by 8% (0.10 m/s; *p* = 0.001) and metabolic rate decreased by 19% (*p* = 0.007), resulting in a 25% decrease in energetic cost of transport (*p* = 8*e*-4) compared to walking with exoskeletons applying zero torque. Compared to younger participants in studies optimizing a single objective, our participants required lower exoskeleton torques, experienced smaller improvements in energy use, and required more time for motor adaptation.

**Conclusions:**

Our results confirm that exoskeleton assistance can improve walking performance for older adults and show that multiple objectives can be simultaneously addressed through human-in-the-loop optimization.

**Supplementary Information:**

The online version contains supplementary material available at 10.1186/s12984-023-01287-5.

## Background

Walking can become more challenging with age. 40% of Americans age 65 and older report difficulty with walking or stair climbing [1], with comfortable and maximum gait speeds declining by 10–30% with age [2]. Apart from being a marker of poorer health, slow walking speeds can be prohibitive for navigation of everyday environments [3] and social participation [4]. Walking also becomes more energetically taxing with age. Older adults’ metabolic energy consumption while walking is approximately 20% higher than that of younger adults [5, 6]. Metabolic cost of transport, a measure of energy consumption per distance traveled, is similarly elevated with age by around 30% [7]. Enabling older adults to walk comfortably at faster speeds with reduced energy consumption could help them navigate everyday environments with increased ease, independence, and satisfaction. Making walking easier may further promote physical activity for aging populations, which has numerous positive outcomes, including increased social connectivity, improved cardiovascular health, better cognition, reduced fall risk, and increased life expectancy [8].

Lower-limb exoskeletons have demonstrated potential to make walking easier. Mechanical power provided by an exoskeleton can offload muscle activity and augment biological joint power, enabling increased self-selected walking speed [9–12] or decreased metabolic energy consumption [13–18]. Whole-leg exoskeletons may provide the largest improvements to walking performance [18], but devices that assist at a single joint may be more efficient with respect to weight, size, and cost, making them more practical for everyday use. Among single-joint configurations, ankle assistance provides the largest improvement in energy economy [18].

Exoskeletons have been used to assist older adult gait with promising results. In recent studies, older adults have experienced a 16% increase in walking speed during short bouts [9] and 3–12% reductions in metabolic energy consumption [9, 14, 15] with single-joint assistance. Greater benefits might yet be possible; a systematic study of control techniques could identify the full potential of exoskeleton assistance.

Personalized exoskeleton assistance may provide the greatest benefits in walking performance. Personalized assistance results in approximately 2 times the speed improvement [11, 19] and 1.3 to 2 times the metabolic improvement [16, 19] of generically ascribed assistance. Of personalization methods [20–25], human-in-the-loop optimization of powered exoskeleton emulators has demonstrated the largest changes in speed and energy consumption [11, 16, 18]. Human-in-the-loop optimization [20] is a method of estimating exoskeleton characteristics that maximally benefit a given metric of user performance. Powered exoskeleton emulators [26–28] allow for rapid testing of a wide range of these exoskeleton characteristics during walking. Human-in-the-loop optimization of an ankle exoskeleton emulator resulted in a 42% increase in walking speed [11] and a 39% reduction in metabolic rate [16] for younger adults, aged 22–33 years.

Optimization of exoskeleton assistance has yet to be tested with older adults. Given the increase in health heterogeneity with age, particularly in measures of mobility and frailty [29], personalized assistance may be more comfortable and feasible than generic assistance for older exoskeleton users. Human-in-the-loop optimization of exoskeleton assistance should be tested with older adults.

Multi-objective exoskeleton optimization is a critical next step. While some studies have assessed the benefit of exoskeletons on speed and energy consumption simultaneously [11, 19], none have optimized exoskeleton assistance for both measures. Targeting multiple objectives can make optimization harder due to a more complex cost landscape. Physiologically, it may also be difficult to achieve simultaneous improvements in an increasing number of objectives. With exoskeleton assistance optimized for speed alone, metabolic energy cost naturally varied between 31% lower to 78% higher than in normal walking [11]. With exoskeleton assistance optimized for energy cost alone, benefits were distributed between speed and energy cost during free gait [19]. Energy savings were approximately half of those observed at a fixed speed [16, 19] and speed improvements were more modest [19]. The efficacy of human-in-the-loop optimization for multiple objectives has yet to be established.

Extensive training is required for users to achieve the full benefits of exoskeleton assistance. Walking performance is quickly improved upon use of an exoskeleton, but further improves as people better learn to walk with the device [14, 16]. During initial stages of learning, movement variability is heightened as the nervous system explores candidate control strategies; movement variability then decays exponentially with experience as the nervous system refines and exploits an optimal control strategy [30–33]. Healthy younger adults require approximately three hours of training to fully adapt to ankle exoskeleton use [16, 32]. Age may increase the time needed to learn new motor tasks [34], so longer protocols may be required for older adults to fully adapt to the same devices. Characterizing older adults’ motor learning while walking with exoskeletons would enable the development of more effective experimentation and prescription protocols.

Self-pacing treadmill controllers have been developed to study natural gait patterns in a laboratory setting. Traditionally, laboratory-based gait studies require participants to walk at a fixed speed or to update their speed through manual control of a treadmill. Self-pacing treadmill controllers instead enable a force-instrumented treadmill to continuously adapt to a participant’s speed while walking [35–37]. Younger adults perceived this method of speed selection to be as comfortable and easy as conventional treadmill control [37]. Further, their speed on the self-paced treadmill more accurately reflected overground walking speed than conventional treadmill speed selection [37]. Older adults might experience self-paced treadmill control differently than younger adults; this approach should be validated among older participants.

The primary objectives of our study were to: (1) assess the extent to which trained older adults can garner speed and energy benefits from ankle exoskeleton assistance and (2) extend human-in-the-loop optimization to target both self-selected walking speed and metabolic energy consumption in a multi-objective paradigm. We also aimed to assess the efficacy of our self-pacing treadmill controller for use in older adult gait studies, characterize older adults’ adaptation to exoskeleton use, and gain initial insights into the effects of aging on response to exoskeleton assistance. To this end, ten healthy older adults walked on a self-paced treadmill with exoskeleton emulators that assisted ankle plantarflexion. We used multi-objective human-in-the-loop optimization to search for exoskeleton parameters that resulted in the greatest benefits in speed and metabolic rate for each participant, then assessed participant responses after four hours of training and optimization. We compared our results to prior human-in-the-loop studies with younger adults to discern differences across age groups. We expected that older adults would adapt to exoskeleton and self-paced treadmill use throughout the study, ultimately achieving distributed benefits in self-selected walking speed and metabolic rate, resulting in large reductions in metabolic cost of transport.

## Methods

**Fig. 1:**
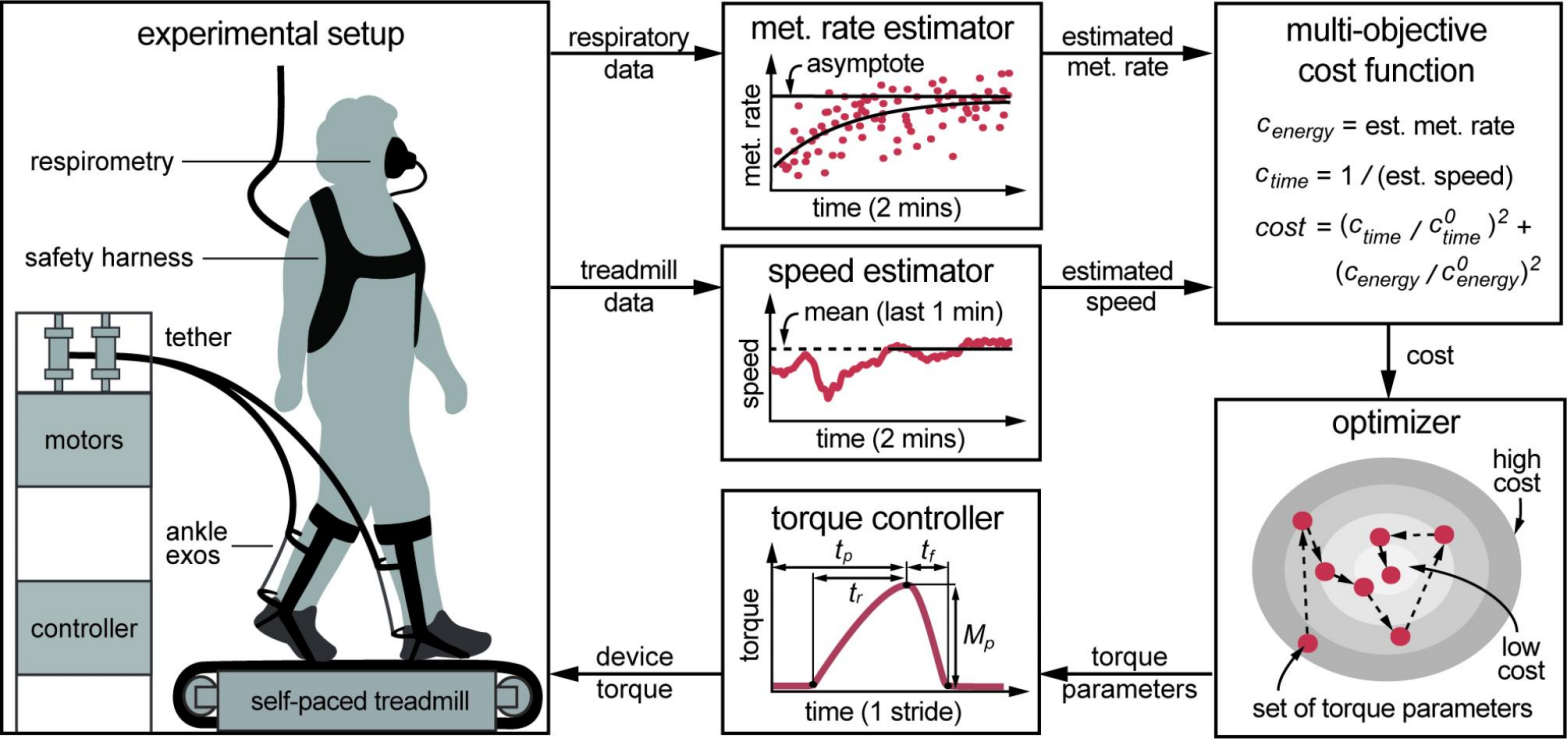
Multi-objective human-in-the-loop optimization. Real-time speed and metabolic (met.) data are collected as a participant walks on a self-paced treadmill wearing bilateral tethered ankle exoskeletons (exos). These data are used to evaluate exoskeleton torque parameters according to a multi-objective cost function. An optimizer generates new sets of exoskeleton parameters to be tested, in search of lower-cost parameters. Each parameter set – peak magnitude (*M*_*p*_), peak time (*t*_*p*_), rise time (*t*_*r*_), and fall time (*t*_*f*_) – defines an exoskeleton torque profile that is applied once per step during walking

Participants aged 65 and older walked on a self-paced treadmill wearing an indirect calorimetry device and ankle exoskeleton emulators that provided an assistive plantarflexion torque once per step (Fig. 1). A human-in-the-loop optimizer varied torque profiles every two minutes in search of parameters that maximized the user’s self-selected walking speed and minimized the user’s metabolic rate according to a multi-objective cost function. Participants were instructed to walk at a comfortable speed during all bouts. After four hours of training and optimization, a validation session was conducted to compare self-selected speed, metabolic rate, metabolic cost of transport, optimized exoskeleton mechanics, and spatiotemporal gait parameters between unassisted and assisted walking conditions.

### Exoskeleton hardware and control

Participants wore an ankle exoskeleton emulator on each leg. The exoskeleton [26] consisted of a lightweight frame embedded into the sole of a commercially available running shoe. Participants donned the exoskeleton via the shoe and a padded strap secured below their knee. Exoskeletons were instrumented with a rotary encoder for angle measurement at the ankle joint and a strain gauge for torque measurement at a heel spur on the exoskeleton frame. Each exoskeleton was actuated by a single off-board motor (Humotech). A flexible Bowden cable transmitted mechanical power from the motor to the heel spur, such that motor force generated a plantarflexion torque about the ankle joint [38]. A real-time computer (Speedgoat Performance) controlled the timing and magnitude of torque delivery. This design allowed the emulators to assume a range of exoskeleton characteristics, facilitating the discovery of optimal assistance strategies for many users without the need to develop new hardware [38].

We used the ankle exoskeleton emulators to test a wide range of assistive plantarflexion torques. Prior research has demonstrated the efficacy of exoskeleton emulators to rapidly and precisely test many assistive torque patterns [20, 22]. A time-based controller [11, 16, 20] was used to produce desired torque trajectories. This technique can generate exoskeleton torques similar to biological joint torques with adjustable timing and amounts of applied positive joint work. Exoskeleton torque trajectories were parameterized by peak magnitude (*M*_*p*_), peak time (*t*_*p*_), rise time (*t*_*r*_) and fall time (*t*_*f*_) and smoothed by a cubic spline between parameterized nodes (Fig. 1). Timing parameters were set as percentages of stride time, which was calculated from a running average of seconds elapsed between ipsilateral heel strikes. Based on prior studies [11, 16, 20], parameters were constrained to 10 N-m ≤ *M*_*p*_ ≤ 60 N-m, 45% ≤ *t*_*p*_ ≤ 55%, 10% ≤ *t*_*r*_ ≤ 40%, 5% ≤ *t*_*f*_ ≤ 20% to ensure participant safety. Torque profiles within our parameterized range have been previously shown to benefit speed and metabolic energy consumption for healthy younger adults, aged 22–37 years [11, 16, 20].

### Self-paced treadmill

We used a self-pacing controller to enable participants to continuously modulate their walking speed on a treadmill, as they would overground. Force-plate data from an instrumented treadmill (Bertec) was used to calculate the participant’s walking speed in real time. The treadmill belt smoothly transitioned toward this pace once per footstep [37]. Participants were instructed on how to modulate the treadmill’s speed via their own speed and position on the device. Prior work has validated this controller’s adaptability to participant behavior and reliability in measuring self-selected walking speed on healthy younger adults [37].

We instructed participants to walk at a comfortable pace in order to capture their self-selected walking speed. Given that instructions can strongly influence the speeds people self-select [39], we instructed participants to “walk at a comfortable pace” for all bouts of walking, where “a comfortable pace means walking naturally as you do in your everyday life, similar to how you walked into our lab today or how you would stroll around the mall as you are casually browsing.”

### Multi-objective human-in-the-loop optimization

We used human-in-the-loop optimization to search for exoskeleton torque parameters that increased self-selected walking speed and decreased metabolic energy consumption (Fig. 1). A multi-objective cost function was defined to target simultaneous speed and energy benefits: $cost=({c}_{time}/{c}_{time}^{0}{)}^{2}+({c}_{energy}/{c}_{energy}^{0}{)}^{2}$. Time cost (${c}_{time}$), the time taken to travel a unit distance, was calculated as the inverse of self-selected speed during a walking trial. Net metabolic rate, or energy cost (${c}_{energy}$), was calculated as the difference between steady-state metabolic rate during a walking trial and resting metabolic rate from the same session. Nominal time cost (${c}_{time}^{0}$) and nominal energy cost (${c}_{energy}^{0}$) were calculated from the participant’s first session as they walked in normal shoes. We normalized each cost term relative to nominal values to target relative speed and energy improvements for each participant and to allow for a comparison of costs between participants. Normalized cost terms were squared to target improvement in both measures, ensuring that we did not favor a large benefit to one term with a penalty to the other. Squared terms further prioritized reducing larger costs over reducing smaller costs. The relative importance older adults place on speed and energy consumption while walking with exoskeletons is unknown, but we expected that participants would prefer balanced improvements in both measures, and so we weighted time cost and energy cost terms equally.

We considered several alternatives when designing this cost function. We hoped to guide balanced improvements in speed and metabolic rate that would further benefit metabolic cost of transport. We were interested in metabolic rate in addition to metabolic cost of transport because it is a marker of perceived exertion and proximity to one’s aerobic threshold [40]. We theorized that a single cost of transport term would not well-encode speed improvements; dual cost of transport and ${c}_{time}$ terms would over-weight speed improvements; dual cost of transport and ${c}_{energy}$ terms would over-weight energy improvements, but that dual ${c}_{time}$ and ${c}_{energy}$ terms would equally weight speed and energy improvements while also improving cost of transport. For this reason, we selected the last approach.

Our optimization algorithm iteratively searched for exoskeleton parameters that resulted in lower costs. We used a covariance matrix adaptation evolutionary strategy [41] for optimization. In this approach, a set of parameters (values for *M*_*p*_, *t*_*p*,_*t*_*r*_, *t*_*f*_) are generated and subsequently evaluated by calculating $cost$ during two minutes of walking with the associated torque profile. Costs for each set of parameters are used to generate new sets of parameters for testing. We repeated this process for 96 unique parameter sets, or 192 min of walking, per participant. Prior studies have demonstrated that approximately 130 min of walking is sufficient to determine optimized ankle exoskeleton parameters for younger adults [16, 18]. We designed our optimization protocol to be one hour longer than this estimate to accommodate possible age-related differences in motor learning without unduly increasing the time burden for participants.

### Participants

Ten community dwelling older adults were recruited for this study (Table 1, 5 females and 5 males, age: 72 ± 3 yrs, body mass: 77.3 ± 18.9 kg, height: 1.71 ± 0.08 m). Inclusion criterion was age of at least 65 years. Exclusion criteria were prior exoskeleton experience and pre-existing orthopedic or cardiovascular conditions. All participants provided written informed consent before participation. The study protocol was approved by the Stanford University Institutional Review Board.

**Table 1:** Participant demographics

Participant	Sex	Age (yrs)	Mass (kg)	Height (m)	Overground Speed (m/s)
1	F	71	77	1.73	1.39
2	M	67	85	1.79	1.51
3	M	75	104	1.65	1.21
4	M	70	79	1.80	1.30
5	F	75	67	1.70	1.14
6	F	73	53	1.57	1.24
7	M	72	81	1.75	1.31
8	M	69	110	1.80	1.40
9	F	75	61	1.65	1.01
10	F	68	56	1.65	1.52
Mean ± SD	5 F, 5 M	72 ± 3	77.3 ± 18.9	1.71 ± 0.08	1.30 ± 0.16

The number designation for each participant is consistent across text, tables, and figures.

### Experimental protocol

Participants followed a six-day experimental protocol during which they walked on a self-paced treadmill wearing either ankle exoskeletons or normal running shoes identical to those used in the exoskeletons. The protocol consisted of a familiarization session to acclimate to the treadmill and exoskeletons, four human-in-the-loop optimization sessions to test a total of 96 unique torque profiles, and a validation session to assess walking performance with optimized assistance. Participants fasted for two hours before each session to mitigate the thermic effect of food on measurements of metabolic rate. Each session was typically followed by one to four rest days. This rest period was intended to allow participants to recover from potential fatigue and avoid losses in learning retention. Participant 4 had two consecutive sessions due to scheduling needs. Participant 5 had a three-week rest period due to hardware malfunction and participant illness unrelated to the study. We verified that this longer rest did not substantially impact adaptation based on spatiotemporal gait data. One participant ended the study after five days because optimization appeared to have converged.

Throughout each session, participants were instructed to walk at a comfortable pace, with an explanatory script read to ensure consistent interpretation of instructions: “A comfortable pace means walking naturally as you do in your everyday life, similar to how you walked into our lab today or how you would stroll around the mall as you are casually browsing. We do not want you to view this as active exercise where you are trying to get your heart rate up. We do not want you to walk slowly in the name of saving energy for later. We do not want you to speed up if you are excited about being close to the end of the session. We want you to keep the mindset of walking comfortably every time you are walking for this study. We recognize that what speed feels comfortable can be different throughout the session, for instance, as you get different types of assistance from the exoskeletons – that is okay. We want you to walk however feels the most natural at that moment, with the mindset of walking comfortably.“

#### Familiarization session

Participants first practiced modulating their speed on the self-paced treadmill while wearing normal shoes. Most participants reported that they were comfortable using the self-paced treadmill after about 15 min, though one required about one hour. Once participants were comfortable modulating their speed on the self-paced treadmill, nominal time cost (${c}_{time}^{0}$) and nominal energy cost (${c}_{energy}^{0}$) were measured from six minutes of normal walking on the treadmill. Participants then donned the exoskeletons and walked with torque profiles that broadly sampled the parameter space. This included a generic torque profile (*M*_*p*_ = 0.54 × body mass, *t*_*p*_ = 53%, *t*_*r*_ = 26%, *t*_*f*_ = 10%) that was found to be well-tolerated and beneficial in prior studies [11, 16, 20], a torque profile with a short rise time (*t*_*r*_ = 15%), a long rise time (*t*_*r*_ = 40%), and an early peak time (*t*_*p*_ = 40%). For each assisted bout, *M*_*p*_ was initially set to 1/3 × 0.54 × body mass and was increased incrementally based on participant comfort. A bout ended when either (1) participants indicated that a larger torque magnitude would be uncomfortable, (2) *M*_*p*_ reached 3/2 × 0.54 × body mass, or (3) *M*_*p*_ reached 60 N-m.

#### Optimization sessions

Human-in-the-loop optimization was conducted for 96 unique candidate torque profiles, distributed evenly over four sessions. Each session was divided into three 20-minute sets. A set consisted of a short warm-up, one generic assistance profile, and eight unique torque profiles. Generic assistance was repeated in each set to track motor adaptation. The first set of parameters were initialized around generic timing parameters and an *M*_*p*_ equal to the smaller value of either 0.54 × body mass or 2/3 of the maximum tolerated magnitude from familiarization. The upper limit of *M*_*p*_ was set to the maximum tolerated magnitude from familiarization. To accommodate potential for adaptation and increased comfort with exoskeletons, this limit was increased by 3 N-m – up to the 60 N-m safety limit – if the generation’s mean *M*_*p*_ was within 3 N-m of the existing limit. Participants were given the option of a break after ten minutes of consecutive walking. At the end of each 20-minute set, a minimum break of three minutes was required to mitigate fatigue. Participants could skip any condition if deemed uncomfortable. Total walking time for each optimization session was approximately one hour.

#### Validation session

Participants completed a double-reversal validation session with a timed 10-meter walk test overground (OG) and three conditions on the self-paced treadmill: walking in normal shoes (NS), walking with the exoskeletons providing zero torque (ZT), and walking with the exoskeletons providing optimized torque (OT). In the zero-torque condition, motors were controlled to track the ankle joint so as to maintain a set amount of slack in the rope of the tether. Exoskeleton strain gauges were used to verify that applied torque during this condition was negligible. In all trials, participants were instructed to walk at a comfortable pace. Speed was measured during all trials. Metabolic data were collected during all trials on the self-paced treadmill. Six minutes of quiet standing (QS) were conducted to capture the participant’s resting metabolic rate before reversed order trials (OG-NS-ZT-OT-QS-OT-ZT-NS-OG). Generic assistance was not tested in the validation session in consideration of how much walking older adults could comfortably complete in one session and to prioritize the accuracy of measures central to the primary hypotheses.

### Measured outcomes

We measured self-selected walking speed from treadmill belt speed and computed metabolic rate from respiratory data. Self-selected walking speed (m/s) was calculated as the average treadmill speed in the latter half of each trial. An indirect calorimetry device (Cosmed Quark CPET) was used to measure participants’ breath-by-breath volumetric O_2_ consumption and CO_2_ production. Metabolic rate was computed using a standard equation [42]. During optimization sessions, a first-order dynamical model was fit to two minutes of metabolic rate data to estimate steady-state metabolic rate for each condition. This approach has previously been found to result in low estimation errors (approximately 4%) compared to averaging six minutes of metabolic data [20]. Metabolic rate during a validation session was calculated as the average rate from the final three minutes of a six-minute trial. Metabolic cost (W/kg) was calculated as the participant’s net metabolic rate during a walking condition, normalized to body mass. Metabolic cost of transport (J/kg/m) was calculated in post-hoc analysis as the quotient of metabolic cost by self-selected walking speed.

Exoskeleton torque and ankle angle were measured by on-board sensors. Exoskeleton ankle angular velocity was calculated from numerical differentiation of ankle angle. Exoskeleton power was calculated as the product of torque and ankle angular velocity. Torque, angle, and power profiles were calculated from the last three minutes of validation trials by segmenting data into strides, then averaging across strides. Reported torque and power measures were normalized to participant body mass.

Force data from an instrumented split-belt treadmill were used to determine step length, step width, stance time, stride time, step frequency, and step frequency variability from the final three minutes of validation trials. Ground reaction forces were low-pass filtered with a cutoff frequency of 15 Hz (third-order Butterworth). Heel strike and toe-off events for each foot were identified from the vertical ground reaction force of the respective treadmill belt crossing 50 N. Step length and width (m) were calculated from center of pressure locations of contralateral heel strikes. Stance time was measured as seconds elapsed between ipsilateral heel strike and toe-off events. Stride time was measured as seconds elapsed between ipsilateral heel strikes. Stance duration (%) was calculated as the quotient of stance time by stride time. Step frequency variability was calculated from the standard deviation of step frequency after high-pass filtering with a 0.033 steps^-1^ cutoff, as described in [32]. De-trended step frequency variability was calculated from the methodologies described in [43]. The component of step frequency (*f*) related to speed (*v*) was calculated by the equation $f=\alpha *{v}^{\beta }$ where *α* and *β* were estimated for individual trials using non-linear least squares. We subtracted this component from step frequency and took the standard deviation to yield de-trended step frequency variability.

### Statistical analysis

Linear regression was used to investigate the relationship between participant speed during normal walking overground and on our self-paced treadmill. A linear model (${v}_{SPT}={a}_{0}*{v}_{OG}+{b}_{0}$) was estimated using a least squares algorithm. Pearson’s linear correlation coefficient ($R$) was reported, where 1 and 0 correspond to a perfect and no correlation, respectively. The linear fit correlation value was considered statistically significant for *p* < 0.05.

A one-way random-effects ANOVA and post-hoc paired t-tests with the Bonferroni correction were used to determine the effect of walking condition (normal shoes, zero torque, or optimized torque) on self-selected walking speed, metabolic cost, and cost of transport. This approach accounted for within-person correlation across conditions and variation across participants as a random effect. A one-way random-effects ANOVA and post-hoc paired t-tests were also used to investigate the effect of walking condition on gait parameters. For all analyses, the significance level was *α* = 0.05.

Data from validation sessions were used to calculate the mean and standard deviation of all reported measures. We report percent changes between zero torque and optimized torque conditions as the primary outcomes of this analysis. Comparisons to zero torque isolate the effect of assistance independent of the effect of device mass, while comparisons to normal shoes include device-specific costs. A portable exoskeleton capable of applying the optimized assistance profiles identified in this study would have different device-specific costs than the emulators used (e.g., from added worn mass due to on-board actuation or lower worn mass due to streamlined design for a specialized function). Therefore, changes in outcomes between optimized torque and normal shoes conditions in this study should not be directly interpreted as the benefits users would receive from walking with exoskeletons in the real world. Thus, our study was designed to focus first on the effects of assistance rather than the effects of the exoskeleton emulators.

An exponential model ($y=1+a{e}^{-t/b}$) was fit to step frequency variability data during generic assistance trials, normalized to participants’ baseline variability during normal walking and pooled between all participants. In this model, *t* denotes experience time in minutes, *a* is a scaling factor, and *b* is the time constant. We did not observe a steady-state response within the duration of our study and thus fixed the steady-state response of the exponential model to baseline during normal walking ($y=1$) (32). Variables $a$ and $b$ were estimated using a nonlinear least squares algorithm. Bootstrapping was used to determine the 95% confidence interval of the exponential model (16,32). Residuals were calculated between each data point and the exponential fit of the pooled data. Residuals were sampled with replacement at each time point to simulate ten new participants. A new exponential model was fit to the simulated participants using nonlinear least squares. This process was repeated for 10,000 trials and confidence limits were established from the 25th and 75th percentiles. The median and interquartile range of the time constant were reported.

Linear regression was used to investigate the relationship between participant age and performance outcomes (self-selected waking speed, metabolic cost, and cost of transport), optimized exoskeleton parameters (*M*_*p*_, *t*_*p*_, *t*_*r*_, *t*_*f*_), and biomechanical responses to assistance (peak ankle angle, exoskeleton power, and spatiotemporal gait features). The coefficient of determination was calculated for each linear regression. A linear fit was considered statistically significant for *p* < 0.05.

## Results

### Self-paced treadmill versus overground walking speed

**Fig. 2:**
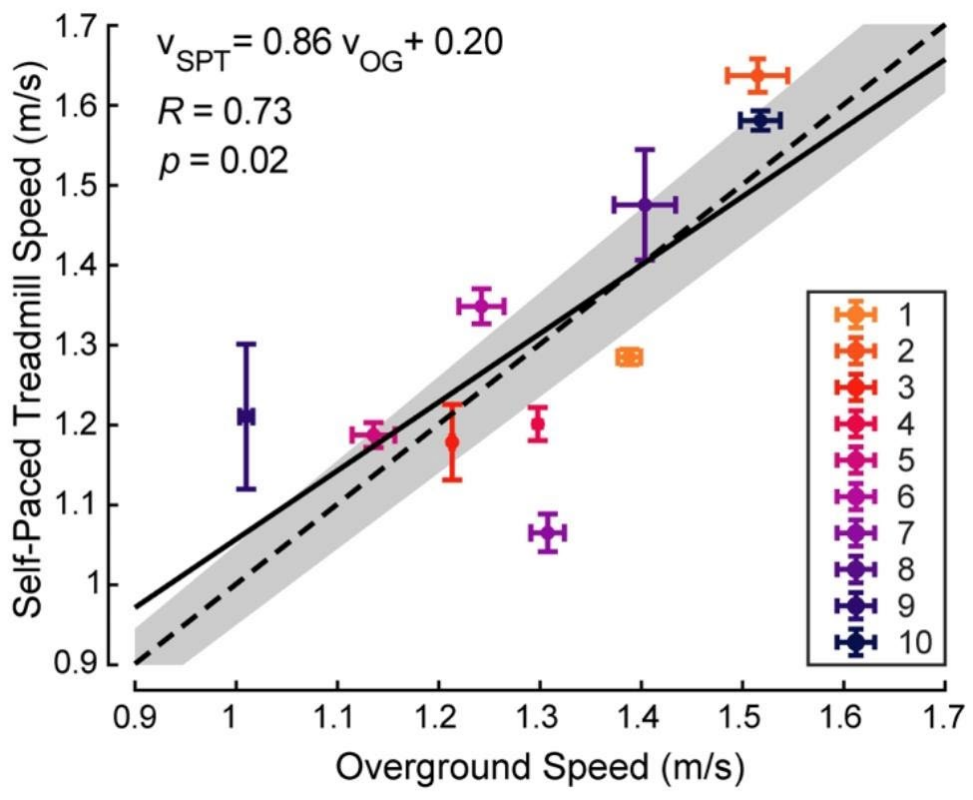
Correlation of speeds measured while walking overground (${\varvec{v}}_{\varvec{O}\varvec{G}}$) and on the self-paced treadmill (${\varvec{v}}_{\varvec{S}\varvec{P}\varvec{T}}$). Colored circles show each participant’s average speed from two overground trials and two self-paced treadmill trials, both while wearing normal shoes. Whisker ends indicate speed in each trial. The linear model (solid), identity line (dashed), and ± 5% from identity (gray shaded region) are shown

Walking speeds measured on the self-paced treadmill were significantly correlated to those measured overground (Fig. 2, *p* = 0.02). Linear regression of experimental data (${v}_{SPT}=0.86{v}_{OG}+0.2$) was similar to the identity line (${v}_{SPT}={v}_{OG}$) and the correlation coefficient was high (*R* = 0.73). Overground speed (1.30 ± 0.16) was not significantly different from treadmill speed (1.31 ± 0.18 m/s) on average (paired t-test, *p* = 0.76). Trial-to-trial differences in walking speed were larger on the treadmill (0.07 ± 0.05 m/s) than overground (0.03 ± 0.02 m/s).

### Self-selected walking speed, metabolic cost, and cost of transport

**Fig. 3:**
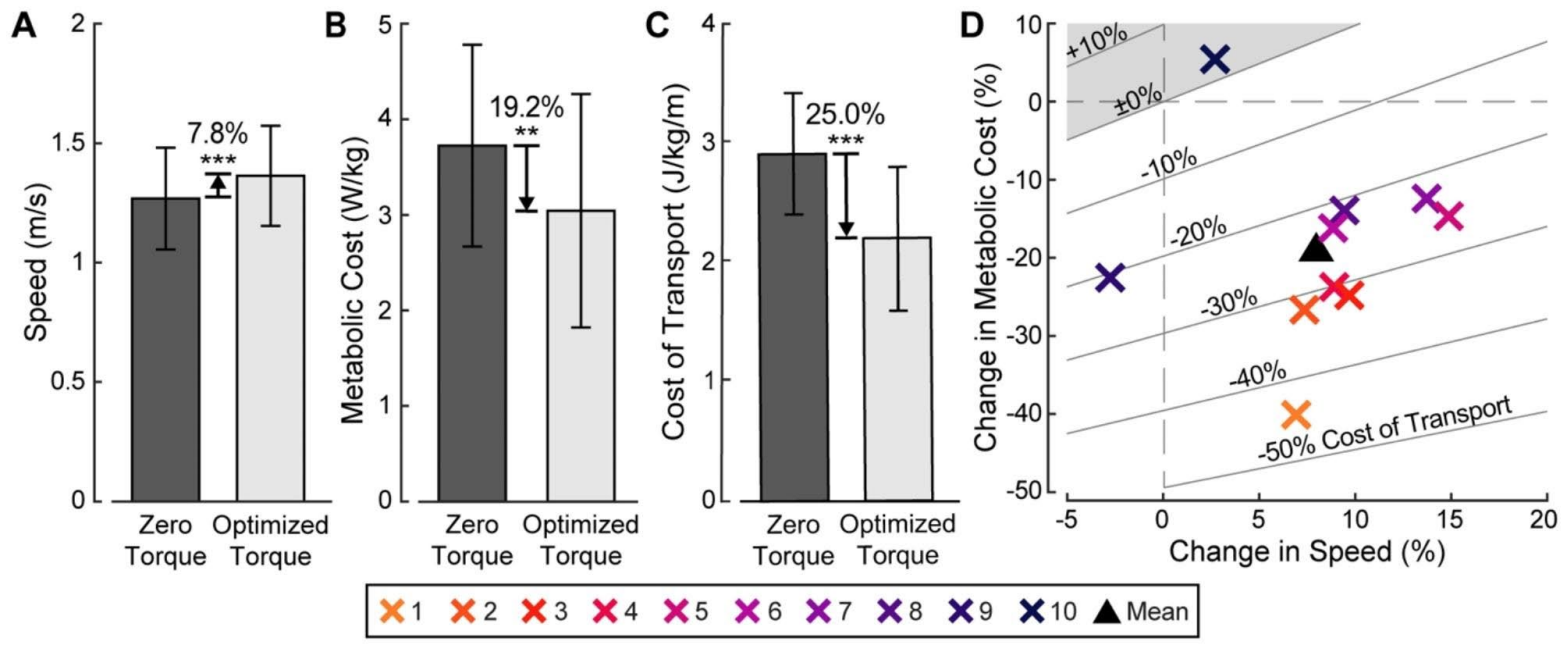
Changes in self-selected walking speed, metabolic cost, and cost of transport with assistance. (**A**) Average increase in self-selected walking speed with assistance. (**B**) Average decrease in metabolic cost with assistance. (**C**) Average decrease in cost of transport with assistance. Comparisons across walking conditions were made using paired t-tests. Error bars show standard deviation. Statistical significance between conditions is denoted with *** for *p* ≤ 0.001 and ** for *p* ≤ 0.01. (**D**) Relative changes in self-selected walking speed, metabolic cost, and cost of transport with assistance. Diagonal lines indicate percent changes in cost of transport. Shaded region represents increased cost of transport with assistance. Participant numbers are ordered from greatest to least percent change in cost of transport

Walking condition had a significant effect on self-selected walking speed, metabolic cost, and cost of transport (ANOVA, *p* = 5.1*e*-4, *p* = 0.003, *p* = 4.8*e*-5, respectively). Self-selected walking speed, metabolic cost, and cost of transport significantly improved when walking with optimized exoskeleton assistance compared to walking with exoskeletons providing zero torque (Fig. 3). Self-selected walking speed increased by 7.8 ± 5.0% (0.10 ± 0.05 m/s) with optimized torque compared to zero torque (paired t-test, *p* = 0.001). Speed changes ranged from − 2.8% to + 14.7% (-0.03 to + 0.16 m/s) for different participants. Metabolic cost decreased by 19.2 ± 12.0% (0.68 ± 0.51 W/kg) with optimized torque compared to zero torque (paired t-test, *p* = 0.007). Changes in metabolic cost ranged from − 40.6% to + 5.5% (-1.42 to + 0.32 W/kg). Cost of transport decreased by 25.0 ± 12.1% (0.71 ± 0.39 J/kg/m) with optimized torque compared to zero torque (paired t-test, *p* = 7.5*e-*4). Changes in cost of transport varied between participants, from − 44.3% to + 3.0% (-1.24 to + 0.11 J/kg/m). Compared to walking in normal shoes, self-selected walking speed increased by 3.6 ± 5.2% (0.05 ± 0.07 m/s), metabolic cost decreased by 12.7 ± 16.0% (0.41 ± 0.69 W/kg), and cost of transport decreased by 15.6 ± 15.6% (0.41 ± 0.48 J/kg/m) with optimized torque (paired t-test, *p* = 0.15, *p* = 0.28, *p* = 0.07, respectively). Absolute changes in self-selected walking speed, metabolic cost, and cost of transport between conditions are shown for each participant in Additional File 1: Figures S1-2.

### Exoskeleton mechanics

**Fig. 4:**
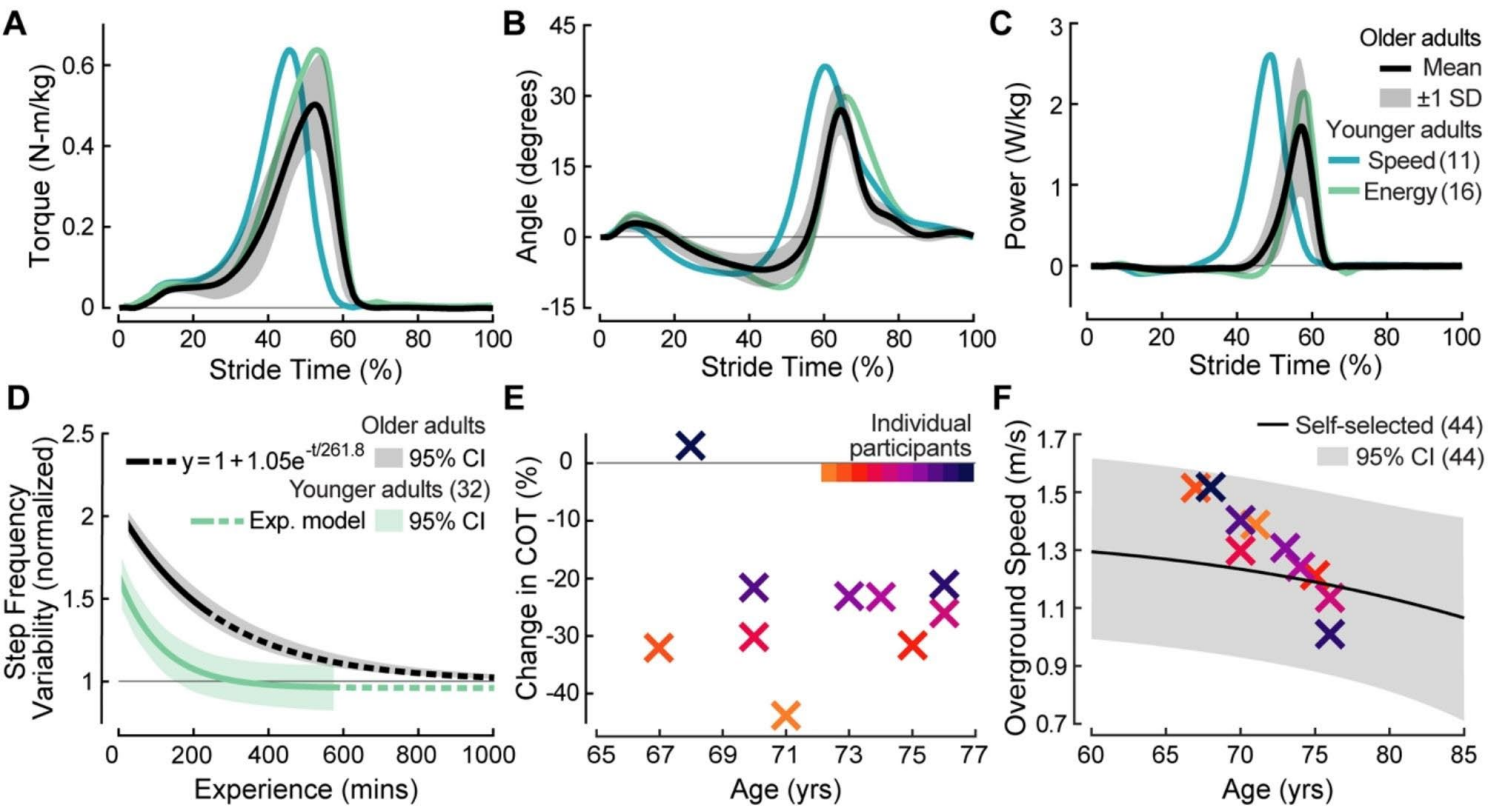
Effects of aging on responses to exoskeleton assistance. (**A**) Measured optimized exoskeleton torque scaled to body mass. (**B**) Exoskeleton ankle angle during assisted walking. Positive angle corresponds with plantarflexion. (**C**) Exoskeleton ankle power, scaled to body mass. (**A**-**C**) Black lines and gray shaded regions show average values and *±* 1 standard deviation for older adults. Blue and green lines show average values for younger adults in human-in-the-loop optimization studies targeting faster walking speed [11] and lower metabolic rate [16], respectively. (**D**) Changes in normalized step frequency variability as participants adapt to exoskeleton assistance. Exponential (exp.) model fits are plotted in solid lines for the study duration and extended to asymptotes by dashed lines. 95% confidence intervals are shaded in gray and green for older and younger adults, respectively. Younger adult trends are recreated from [32]. (**E**) Cost of transport changes between unassisted (zero torque) and assisted walking, observed across participant ages. (**F**) Age-related changes in walking speed. Average self-selected overground speed (black line), and 95% confidence interval (gray shaded region) are recreated from [44]. Older adult participants are plotted in unique colors

Optimized torque was characterized by a peak of 0.54 ± 0.10 N-m/kg at 52.3 ± 1.1% stride, a rise time of 27.3 ± 7.0%, and a fall time of 10.4 ± 2.6% (Fig. 4A). The mean values of the optimized parameters were similar to those of generic assistance (*M*_*p*_ = 0.54 N-m/kg, *t*_*p*_ = 53%, *t*_*r*_ = 26%, *t*_*f*_ = 10%; paired t-test, *p* = 0.98, *p* = 0.07, *p* = 0.59, *p* = 0.45, respectively), though inter-subject variation was high (Additional File 1: Table S1).

Peak plantarflexion changed substantially with assistance (paired t-test, *p* = 9.3*e-*7), while peak dorsiflexion changed minimally (paired t-test, *p* = 0.03). Compared to a peak plantarflexion angle of 11.0 ± 2.5° when walking with zero torque, plantarflexion increased by 148% to 26.5 ± 4.4° with optimized torque (Fig. 4B). Peak dorsiflexion angle was − 10.2 ± 2.5° with zero torque. Dorsiflexion decreased by 10% to a peak angle of -9.2 ± 4.4° with optimized torque (Fig. 4B).

Mechanical exoskeleton power was characterized by a 2.11 ± 0.65 W/kg peak at 57.0 ± 2.2% stride, immediately preceding toe-off (Fig. 4C). Negative power from the exoskeleton was negligible.

### Spatiotemporal gait parameters

Walking condition had a significant effect on step frequency, step frequency variability, and step width (ANOVA, *p* = 0.007, *p* = 8.8*e*-4, *p* = 0.007, respectively), but not step length or stance duration (ANOVA, *p* = 0.90, *p* = 0.16, respectively). Step frequency and step frequency variability increased with assistance. Step frequency was 57.0 ± 5.5 strides/min with zero torque and increased by 8% to 61.7 ± 8.5 strides/min with optimized torque (Additional File 1: Table S2, paired t-test, *p* = 0.01), reflecting the average 8% increase in self-selected walking speed. Step frequency variability increased by 31%, from 0.8 ± 0.2 strides/min with zero torque to 1.0 ± 0.2 strides/min with optimized torque (Additional File 1: Table S3, paired t-test, *p* = 0.004). Other spatiotemporal gait parameters were functionally similar between walking with zero torque and optimized torque. Step length was 0.67 ± 0.09 m with zero torque and 0.66 ± 0.11 m with optimized torque (Additional File 1: Table S4, paired t-test, *p* = 0.80). Step width was 0.18 ± 0.03 with zero torque and 0.20 ± 0.02 m with optimized torque (Additional File 1: Table S5, paired t-test, *p* = 0.02). Stance duration was 61 ± 2% during both zero-torque and optimized torque conditions (Additional File 1: Table S6, paired t-test, *p* = 0.22).

### Motor adaptation

Step frequency variability in response to generic assistance exponentially decayed as a function of experience (Fig. 4, $y=1+1.05{e}^{-t/261.8}$, *R*^2^ = 0.15, *p* = 7.4*e-*73). At the beginning of optimization, participants’ step frequency variability was 1.95 times that of normal walking. Step frequency variability decreased with a time constant of 261.8 min (interquartile range [232.0, 300.2]). Step frequency variability at the end of optimization remained 42% elevated above baseline variability during normal walking. Participants varied step frequency in part through changes in self-selected walking speed. After isolating the component of step frequency independent of speed [43], step frequency variability was closer to baseline values during normal walking (Additional File 1: Figures S4-5).

### Effects of participant age

Participant age did not significantly affect outcomes in this study. Age was not correlated to improvements in speed (*R*^2^ = 0.01, *p* = 0.74), metabolic cost (*R*^2^ = 0.01, *p* = 0.75), or cost of transport (Fig. 4E, *R*^2^ = 0.03, *p* = 0.66) between zero-torque and optimized torque conditions. Age was not correlated to biomechanical responses to assistance (e.g., peak ankle angle: *R*^2^ = 4.3*e-*2, *p* = 0.56). Age was also not correlated to optimized torque parameters (e.g., peak torque magnitude: *R*^2^ = 0.11, *p* = 0.34), except for peak time (*R*^2^ = 0.51, *p* = 0.02). This relationship may be due to younger participants walking at faster speeds with assistance (*R*^2^ = 0.62, *p* = 0.01), where faster speeds are associated with earlier peak times (*R*^2^ = 0.67, *p* = 0.004). An inverse linear relationship between participant age and self-selected walking speed was observed in all tested conditions (e.g., overground: Fig. 4F, *R*^2^ = 0.87, *p* = 8.1*e-*5).

## Discussion

### Responses to exoskeleton assistance

Exoskeleton assistance improved walking performance for older adults. On average, participants experienced an 8% (0.10 m/s) increase in self-selected walking speed, a 19% (0.68 W/kg) decrease in metabolic rate, and a 25% (0.71 J/kg/m) decrease in metabolic cost of transport with optimized ankle plantarflexion torque compared to walking with exoskeletons providing zero torque. These speed and energy benefits have real-world significance. The 0.10 m/s increase in self-selected speed meets the clinical threshold for meaningful change in older adults’ walking performance [45]. Participants’ energy savings were equivalent to consuming only 75% of the energy initially required to travel a given distance or removing a 10 kg backpack during walking [46]. The energy savings further demonstrate promise to mitigate the 20–30% increases in both measures associated with aging [6, 7].

Changes in speed and energy consumption varied between participants (Additional File 1: Figures S1-2). Eight of the ten participants improved in both measures, achieving 6.8–14.7% faster walking speeds, 12.6–40.6% lower metabolic rates, and 21.5–44.3% lower costs of transport when walking with optimized torque compared to walking with zero torque. For two participants, either speed or energy consumption was not improved; as a result, one experienced a slight increase in cost of transport. There are several potential explanations to the variability observed. Self-selected walking speed is easily influenced by contextual or psychological factors [39, 47], which may have affected participants to different extents. Results may also have varied due to participants interacting differently with the optimizer. For example, some participants may have productively explored many gait strategies in response to new exoskeleton torque profiles, while others may have prioritized consistent gait behavior. Individual differences in physical traits such as reduced range of motion may have further led people to interact with the exoskeletons differently. Future studies should explore which characteristics, if any, predict better responses to exoskeletons and what protocols may maximize each user’s benefit from assistance.

The variation in optimized exoskeleton parameters suggests the importance of personalizing assistance. Mechanical exoskeleton power that resulted from optimization varied substantially between participants (Additional File 1: Figure S3, peak of 2.11 ± 0.65 W/kg at 57.0 ± 2.2% stride). While some studies assert that greater exoskeleton power drives larger energetic benefits [13, 48, 49], the range of power magnitudes in this study suggest a more complex human-exoskeleton interaction underpinning metabolic rate [16, 19]. Variation in exoskeleton power was primarily attributed to variation in optimized torque magnitude (0.54 ± 0.10 N-m/kg), though optimized timing parameters also differed among participants (Additional File 1: Table S1 and Figure S3). Rise time was highly varied (27.3 ± 7.0%), suggesting that the parameter is either subject-specific or weakly affects time and energy costs [50]. Peak time and fall time were more consistent between participants (*t*_*p*_ = 52.3 ± 1.1% and *t*_*f*_ = 10.4 ± 2.6%), indicating that exoskeleton torque peaking near the end of stance and quickly falling to zero at toe-off was widely beneficial. It is unknown if these parameters were truly optimal for participants; verifying optimality experimentally would be extremely time-intensive, but prior work demonstrates that human-in-the-loop optimization identifies torque profiles more beneficial than others tested in validation [11, 16].

Step frequency changed between optimized torque and zero-torque conditions, but other spatiotemporal gait parameters were unchanged. Exoskeleton assistance led to a change in self-selected walking speed, which is expected to result in changes to both step frequency and step length. All participants increased their step frequency with optimized torque compared to zero torque (Additional File 1: Table S2). Most, but not all, participants increased step length with assistance such that on average, step length was unchanged between optimized torque and zero-torque conditions (Additional File 1: Table S4). Step width and stance duration were unchanged between optimized torque and zero-torque conditions. These results suggest that gaining benefits from exoskeleton assistance did not require changes to gait that could compromise older adults’ comfort and stability.

Participants exhibited partial motor adaptation in response to exoskeleton assistance. The time progression of step frequency variability was previously identified as an indicator of motor learning, where high variability corresponds to ongoing exploration of motor control strategies [32]. Older adults’ step frequency variability during assisted walking was initially 95% higher than that of normal walking. Step frequency variability then decayed exponentially as a function of experience until the end of optimization, where it remained 42% elevated above baseline (Fig. 4D). Per models of motor learning [30–32], the decrease in step frequency variability indicates that participants adopted an improved control strategy with experience, but its final elevation suggests that participants did not fully adapt to walking with exoskeletons.

Subjective responses to exoskeletons were not formally assessed in this study, but participants were encouraged to share any feelings of discomfort pertaining to exoskeleton use. Participants occasionally skipped exoskeleton conditions during optimization due to discomfort. Two participants reported toe discomfort during conditions that involved walking with greater ankle plantarflexion, likely due to nearing the upper limit of their toe range of motion. A stiffer shoe in the exoskeleton might be sufficient to ease or resolve this issue in future designs. One participant reported mild ankle aching and knee pain after a session, which passed after a rest day. Some discomfort was psychological in nature: participants found it difficult to adjust to the sensation of sharing control over their gait behavior with the exoskeletons. Most participants commented on their improved sense of comfort with the devices by the end of training, with some further volunteering their preference to walk with the exoskeletons over normal shoes during the validation session.

### Effects of aging on responses to exoskeleton assistance

It is unclear whether older and younger adults have different capacities to change their walking speed and energy consumption with exoskeleton assistance. Older adults benefited less from exoskeletons than younger adults did in similar studies [11, 16, 20]. Age-related neuromuscular changes such as reduced muscle strength can decrease maximum gait speed and increase minimum energy consumption during walking [5, 6, 51]. Limitations associated with comfort may also be introduced with age. For example, increased joint pain and stiffness could reduce one’s capacity to plantarflex while wearing an exoskeleton, which may limit speed and energy benefits. Speed and energy changes likely differ between studies in part due to our multi-objective approach distributing benefits between both measures [19]. A single-objective human-in-the-loop study on older adults or a multi-objective human-in-the-loop study on younger adults could help isolate the effect of age on capacity to benefit from exoskeleton assistance.

Optimized exoskeleton assistance differed between older and younger adults. Older adults optimized to smaller torque magnitudes than younger adults did in prior studies (Fig. 4A, older adults: *M*_*p*_ = 0.54 ± 0.10 N-m/kg, younger adults: *M*_*p*_ = 0.69 ± 0.19 N-m/kg or 0.68 ± 0.06 N-m/kg) [11, 16]. Rise time and fall time were similar between age groups. Peak time differed between age groups, but this may be associated with differences in walking speed between studies (older adults: *t*_*p*_ = 52% at 1.36 m/s, younger adults: *t*_*p*_ = 47% at 1.83 m/s or *t*_*p*_ = 54% at 1.25 m/s) [11, 16]. Peak ankle plantarflexion was lower for older adults (27 ± 4°) than for younger adults walking with either speed-optimized or energy-optimized assistance (44° and 32°, respectively) [11, 16]. Older adults’ smaller peak plantarflexion angles could be due to their reduced peak torque magnitudes. Alternatively, a reduced range of ankle motion could have caused participants to optimize to smaller peak exoskeleton torques. In either case, our results suggest that smaller and lighter-weight exoskeletons may be more appropriate for older adults.

Age slowed motor adaptation to walking with exoskeletons. Within 240 min of training, step frequency variability during assisted walking returned to baseline values for younger adults [32], but remained 42% elevated for older adults (Fig. 4D). Elevated step frequency variability could reflect that older adults had increased sensitivity to imperfect exoskeleton torque control or that they were continuing to explore motor control strategies. We estimate that older adults would have taken 797 min to return to baseline step frequency variability while younger adults did so within 229 min (Fig. 4D). With additional training, older adults’ responses to exoskeletons may have been more similar to those observed from younger adults. Long training times may be burdensome to complete in the laboratory, but more feasible with at-home exoskeleton use. Physically active older adults could fully adapt to exoskeleton use in one week, gaining sufficient exposure from normal amounts of daily walking. Less active older adults might accumulate sufficient exposure in a few weeks. The development of portable exoskeletons and targeted training protocols might substantially reduce the barriers to older adults becoming expert exoskeleton users.

Participant age in this study did not affect benefits achieved with exoskeleton assistance. Improvements in self-selected walking speed, metabolic cost, and metabolic cost of transport were observed across ages (Fig. 4E). The size and age range of this sample restricts our ability to establish age-related trends for older exoskeleton users. However, there did not appear to be a relationship between age and optimal exoskeleton parameters nor age and biomechanical responses to exoskeleton assistance within the range of ages tested.

### Self-paced treadmill efficacy

Self-paced treadmills are effective tools to study older adults’ natural walking behavior in a laboratory setting. We observed a strong correlation between overground walking speed and walking speed on a self-paced treadmill (Fig. 2, R = 0.73, *p* = 0.02). A prior study using the same self-pacing controller reported a stronger correlation between these conditions for younger adults (*R* = 0.93, *p* < 1*e-*13) [37]. This discrepancy may be due to older adults having increased sensitivity to changes in environment. Additionally, older adults may have been affected by the inclusion of exoskeletons in our protocol, which were associated with faster walking on the treadmill. Nevertheless, the coherence observed between overground and treadmill speeds demonstrates the efficacy of this self-pacing controller for older adults and suggests that our results may translate to overground walking with exoskeletons.

### Multi-objective human-in-the-loop optimization protocol

Human-in-the-loop optimization can effectively target multiple aspects of walking performance. Our approach identified assistance profiles that substantially improved both speed and metabolic rate terms as well as metabolic cost of transport. Additional objectives should be considered in future exoskeleton research; our approach can be extended to incorporate any number of objectives, provided they can be quantified. Aspects of walking like stability and comfort are more difficult to quantify in experimental work but are critical for older exoskeleton users. Notably, optimal exoskeleton behavior will likely differ for each new objective, creating a more complex cost landscape that is harder to optimize. An objective function with more terms will also require more design choices to appropriately weight each term. These factors should be considered when designing multi-objective techniques for exoskeleton optimization.

Psychological or contextual factors in our protocol may have idiosyncratically affected participant behavior. Participant 10, for example, prioritized increasing their walking speed at the expense of an increased metabolic rate for nearly all the 96 unique assistance profiles tested. Upon completion of the study, participants 8 and 9 shared that they were inclined to walk faster at the end of the validation session, either due to a desire to “outperform” their past behavior or excitement about completing the protocol. Given the session’s double-reversal order, these participants’ speeds in the final zero-torque and normal shoes trials appeared artificially inflated (Fig. 2). Our prompt on walking at a comfortable pace was designed to mitigate these phenomena but appears limited in its effect. This may have adversely impacted the benefits derived from exoskeleton assistance for some participants.

Alternate training strategies may provide greater and more consistent benefits in speed and energy consumption. Participants in this study were not given instructions on how to walk with exoskeletons, except that they should do so at a comfortable speed. Providing targeted coaching or biofeedback could guide participants toward more comfortable or effective gait patterns. Protocol duration could also be varied based on an individual’s rate of motor learning to promote convergence to an optimal control strategy. While this study was designed to characterize older adults’ unbiased responses to exoskeletons, a protocol that is more responsive to participant behavior could be fruitful in developing devices for practical use.

This study was not designed to assess the relative benefits of exoskeleton optimization versus training, nor to determine the most effective type of exoskeleton training for older adults. In prior work with younger adults walking with ankle exoskeletons, energetic benefits were attributed one-quarter to personalization of assistance and one-half to training [16]. A training protocol with moderate levels of variation in device behavior, as was used in this study, was found to be more effective for younger adults’ learning than protocols with low or high levels of variation [16]. More work is needed to characterize these relationships for older adults.

### Limitations

The scope of this study is limited by the size and demographic distribution of our sample. We recruited ten participants due to the exploratory nature of the research and time-intensity of data collection. This number was sufficient to demonstrate speed and metabolic improvements, but insufficient to identify participant characteristics that explain observed responses to exoskeletons because of high inter-subject variability. Future work should explore how characteristics like functional mobility or physical activity level might affect participants’ speed and energetic outcomes, exoskeleton-assisted gait features, and timescales of motor adaptation. People who volunteered for our study had diverse physical characteristics but were all highly active and near the onset of age-related mobility decline (Table 1). Participants’ walking speeds were fast on average for their ages, but spanned a meaningful range (Fig. 4F) [44]. Future exoskeleton research should be conducted on participants with more varied physical activity and mobility levels for results to better reflect the broader population.

Our findings are further limited by the study’s execution in a laboratory setting. This environment, distinct from those in which older adults typically walk, may have made it harder for participants to adapt to exoskeleton use. This is corroborated anecdotally by participants requiring extended familiarization to walk on the self-paced treadmill, and quantitatively by their more varied speeds on the treadmill compared to overground walking (Fig. 2). The laboratory environment may further have introduced psychological influences that altered participant behavior [47], potentially offsetting the benefits of exoskeleton assistance. Real-world impact of exoskeleton use will be better understood from studies conducted in natural environments. Recent work demonstrates that portable exoskeleton emulators can provide comparable benefits during indoor and outdoor walking [19], encouraging the translation of our research to real-world settings.

## Conclusions

Older adults achieved clinically meaningful improvements in self-selected walking speed, metabolic energy consumption, and metabolic cost of transport when walking with optimized ankle exoskeleton assistance. These results point to the potential of portable, commercial exoskeletons to make every day walking easier for older adults. The optimal exoskeleton parameters and biomechanical responses to assistance we observed appeared distinct from those previously established for younger adults. Future studies should more deeply probe the effects of aging on response to exoskeleton assistance. A broader exploration of what personal characteristics correlate to optimal exoskeleton parameters could also aid in the development of commercial and rehabilitative exoskeletons for older adults.

In this study, we demonstrated the effectiveness of multi-objective human-in-the-loop optimization. Future work should extend this approach to other important aspects of older adult gait and explore the relative weightings of each objective for different individuals, demographic groups, tasks, and environments. A deeper understanding is needed of how objective function design affects exoskeleton optimization and training. Our participants faced challenges while learning to walk with exoskeletons and were still actively exploring motor control strategies at the conclusion of the study. Personalized training protocols could be designed to make learning to walk with exoskeletons faster and easier. This may further enhance each individual’s walking performance and facilitate the adoption of exoskeletons for real-world use.

### Electronic supplementary material

Below is the link to the electronic supplementary material.

**Additional file 1: Figure S1** shows self-selected walking speed, metabolic cost, and metabolic cost of transport data during zero-torque and optimized torque conditions for each participant. **Figure S2** shows self-selected walking speed, metabolic cost, and metabolic cost of transport data during normal shoes and optimized torque conditions for each participant. **Figure S3** shows optimized exoskeleton mechanics for each participant. **Figure S4** shows individual participant data of step frequency variability in repeated generic torque conditions. **Figure S5** shows individual participant data of de-trended step frequency variability in repeated generic torque conditions. **Table S1** provides optimized torque parameters for each participant, **Table S2** provides step frequency data (strides/min), **Table S3** provides step frequency variability data (strides/min), **Table S4** provides step length data (m), **Table S5** provides step width data (m), and **Table S6** provides stance duration data (% stride) for each participant during normal shoes, zero-torque, and optimized torque conditions

## Data Availability

Data for this study are available from the corresponding author on reasonable request.

## List of abbreviations

ANOVA: Analysis of variance
c_energy_: Energy cost
${\varvec{c}}_{\varvec{e}\varvec{n}\varvec{e}\varvec{r}\varvec{g}\varvec{y}}^{0}$: Nominal energy cost
CI: Confidence interval
CPET: Cardiopulmonary exercise test
c_time_: Time cost
${\varvec{c}}_{\varvec{t}\varvec{i}\varvec{m}\varvec{e}}^{0}$: Nominal time cost
M_p_: Peak magnitude
NS: Normal shoes
OG: Overground
OT: Optimized torque
SD: Standard deviation
t_f_: Fall time
t_p_: Peak time
t_r_: Rise time
v_OG_: Speed overground
v_SPT_: Speed on self-paced treadmill
ZT: Zero torque
