# Supplementary material for: Optimizing exoskeleton assistance to improve walking speed and energy economy for older adults
Source: J Neuroeng Rehabil. 2024 Jan 2;21:1. doi: 10.1186/s12984-023-01287-5 (PMC10763092; doi:10.1186/s12984-023-01287-5)
Supplement: Supplementary file 1 — Additional file 1: Figure S1 shows self-selected walking speed, metabolic cost, and metabolic cost of transport data during zero-torque and optimized torque conditions for each participant. Figure S2 shows self-selected walking speed, metabolic cost, and metabolic cost of transport data during normal shoes and optimized torque conditions for each participant. Figure S3 shows optimized exoskeleton mechanics for each participant. Figure S4 shows individual participant data of step frequency variability in repeated generic torque conditions. Figure S5 shows individual participant data of de-trended step frequency variability in repeated generic torque conditions. Table S1 provides optimized torque parameters for each participant, Table S2 provides step frequency data (strides/min), Table S3 provides step frequency variability data (strides/min), Table S4 provides step length data (m), Table S5 provides step width data (m), and Table S6 provides stance duration data (% stride) for each participant during normal shoes, zero-torque, and optimized torque conditions [file 12984_2023_1287_MOESM1_ESM.docx]

**Additional File 1 ­– Supplementary Figures & Tables**

**
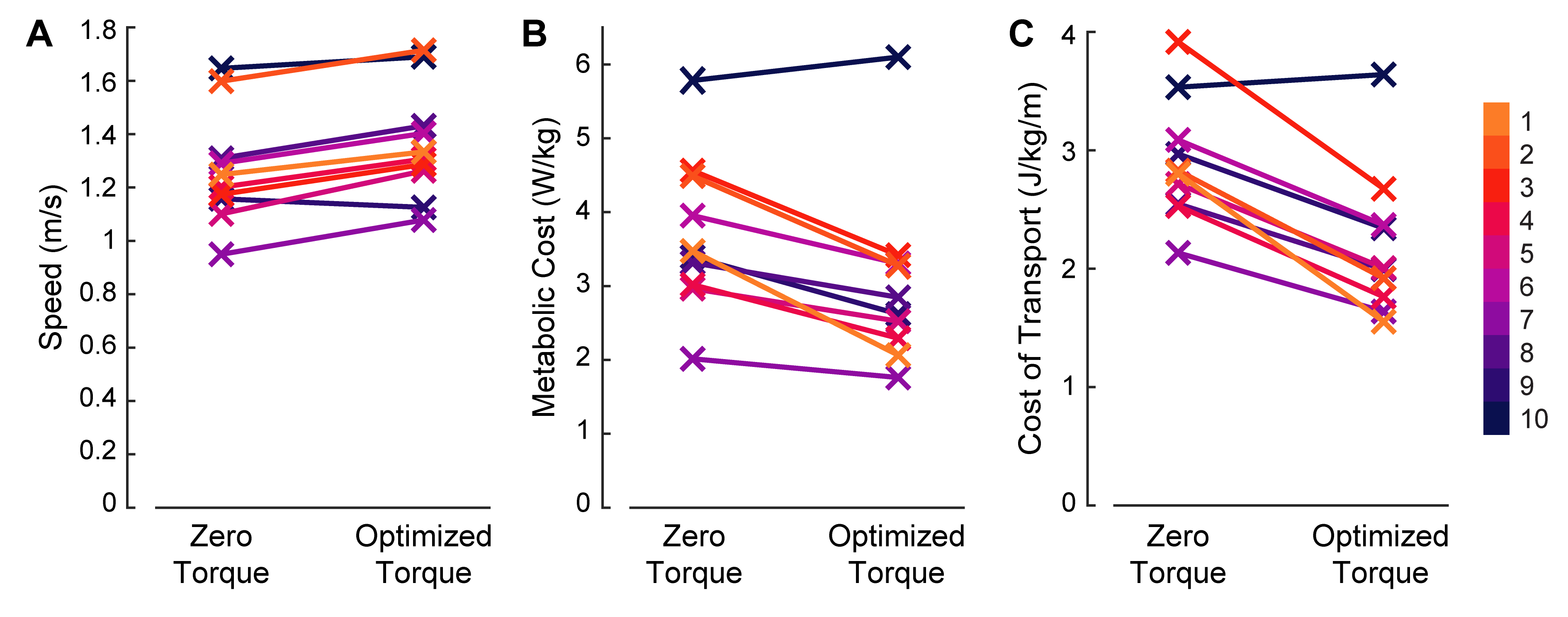
**

**Figure S1. Individual participant data of speed and energetic changes relative to zero torque.** Changes in (A) self-selected walking speed, (B) metabolic cost, and (C) metabolic cost of transport between zero torque and optimized torque conditions.


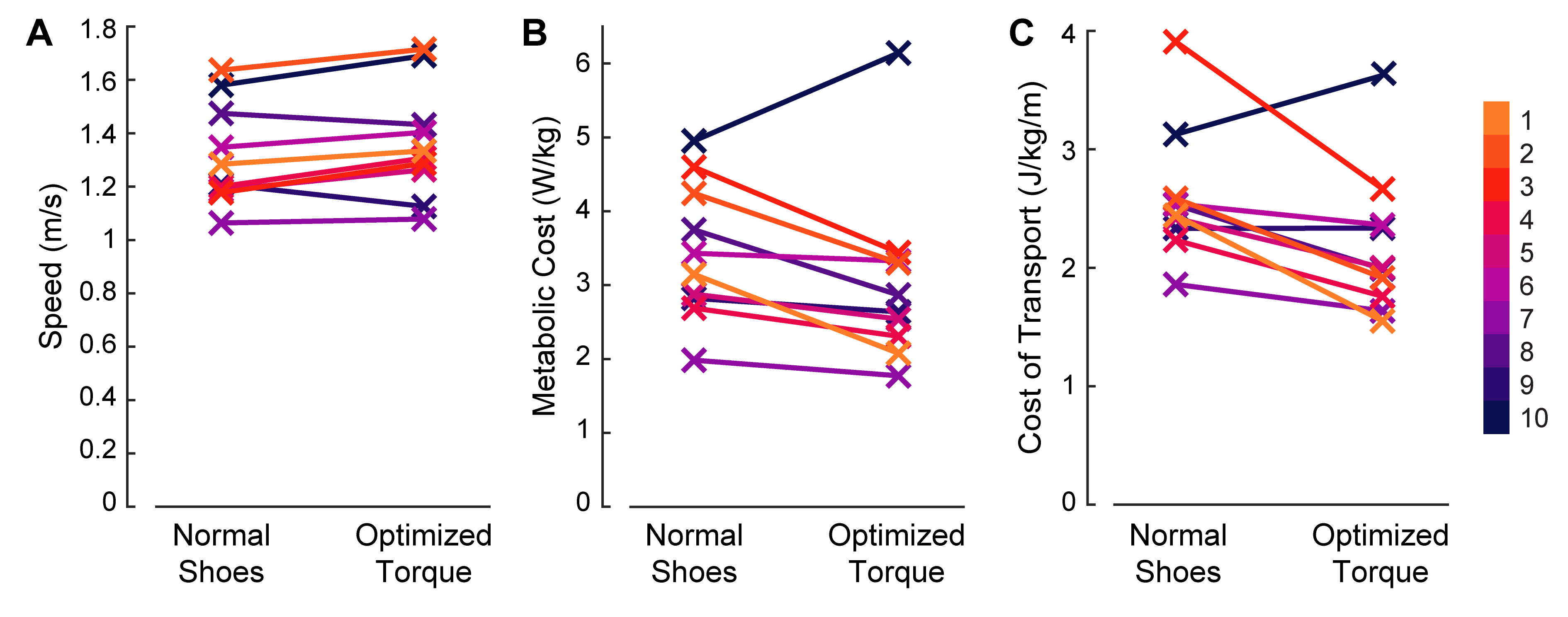


**Figure S2. Individual participant data of speed and energetic changes relative to normal shoes.** Changes in (A) self-selected walking speed, (B) metabolic cost, and (C) metabolic cost of transport between normal shoes and optimized torque conditions.

**­**

**Figure S3. Individual participant data of exoskeleton mechanics.** (A) Measured optimized exoskeleton torque scaled to body mass. (B) Exoskeleton ankle angle during assisted walking. Positive angle corresponds with plantarflexion. (C) Exoskeleton ankle power, scaled to body mass.

**Figure S4. Individual participant data of step frequency variability in repeated generic torque conditions.** Step frequency variability is normalized to baseline variability during normal walking. Exponential model shown in black.

**Figure S5. Individual participant data of de-trended step frequency variability in repeated generic torque conditions.** Step frequency variability is normalized to baseline variability during normal walking. Exponential model shown in black.

| Participant | Peak Torque (N-m/kg) | Peak Time  (%) | Rise Time  (%) | Fall Time  (%) |
| --- | --- | --- | --- | --- |
| 1 | 0.60 | 52.5 | 29.6 | 10.8 |
| 2 | 0.71 | 51.8 | 28.7 | 10.8 |
| 3 | 0.46 | 53.1 | 32.4 | 11.4 |
| 4 | 0.68 | 51.8 | 19.5 | 12.9 |
| 5 | 0.64 | 52.6 | 16.6 | 10.5 |
| 6 | 0.42 | 51.8 | 31.4 | 12.8 |
| 7 | 0.53 | 53.1 | 23.6 | 10.3 |
| 8 | 0.42 | 52.3 | 29.6 | 12.2 |
| 9 | 0.47 | 53.9 | 23.0 | 6.9 |
| 10 | 0.48 | 40.1 | 38.8 | 5.3 |
| Mean ± SD | 0.54 ± 0.11 | 52.3 ± 1.0 | 27.3 ± 6.6 | 10.4 ± 2.5 |

**Table S1. Optimized torque parameters.** Generic torque had a peak magnitude of 0.54 N-m/kg, a peak time of 53% stride, a rise time of 26% stride, and a fall time of 10% stride.

| Participant | Normal Shoes | Zero Torque | Optimized Torque |
| --- | --- | --- | --- |
| 1 | 52.0 | 51.2 | 53.7 |
| 2 | 56.6 | 55.2 | 56.1 |
| 3 | 56.2 | 55.7 | 56.6 |
| 4 | 56.8 | 56.3 | 60.4 |
| 5 | 57.1 | 55.6 | 58.2 |
| 6 | 67.4 | 65.3 | 68.2 |
| 7 | 53.1 | 49.7 | 52.7 |
| 8 | 63.4 | 60.0 | 63.3 |
| 9 | 56.7 | 54.0 | 67.2 |
| 10 | 65.6 | 66.5 | 80.5 |
| Mean ± SD | 58.5 ± 5.2 | 57.0 ± 5.5 | 61.7 ± 8.5 |

**Table S2. Step frequency (strides/min) during normal shoes, zero torque, and optimized torque conditions.**

| Participant | Normal Shoes | Zero Torque | Optimized Torque |
| --- | --- | --- | --- |
| 1 | 0.9 | 1.0 | 0.9 |
| 2 | 1.0 | 0.8 | 0.9 |
| 3 | 0.8 | 0.7 | 0.9 |
| 4 | 0.8 | 0.8 | 1.1 |
| 5 | 0.8 | 0.8 | 1.3 |
| 6 | 1.1 | 0.8 | 1.2 |
| 7 | 0.7 | 0.8 | 1.0 |
| 8 | 0.8 | 0.9 | 1.3 |
| 9 | 0.6 | 0.6 | 1.0 |
| 10 | 0.6 | 0.7 | 0.7 |
| Mean ± SD | 0.8 ± 0.2 | 0.8 ± 0.1 | 1.0 ± 0.2 |

**Table S3. Step frequency variability (strides/min) during normal shoes, zero torque, and optimized torque conditions.**

| Participant | Normal Shoes | Zero Torque | Optimized Torque |
| --- | --- | --- | --- |
| 1 | 0.73 | 0.73 | 0.74 |
| 2 | 0.85 | 0.87 | 0.91 |
| 3 | 0.61 | 0.63 | 0.69 |
| 4 | 0.63 | 0.64 | 0.64 |
| 5 | 0.60 | 0.59 | 0.65 |
| 6 | 0.59 | 0.59 | 0.61 |
| 7 | 0.60 | 0.57 | 0.61 |
| 8 | 0.65 | 0.66 | 0.68 |
| 9 | 0.62 | 0.64 | 0.48 |
| 10 | 0.70 | 0.74 | 0.61 |
| Mean ± SD | 0.66 ± 0.08 | 0.67 ± 0.09 | 0.66 ± 0.11 |

**Table S4. Step length (m) during normal shoes, zero torque, and optimized torque conditions.**

| Participant | Normal Shoes | Zero Torque | Optimized Torque |
| --- | --- | --- | --- |
| 1 | 0.16 | 0.17 | 0.19 |
| 2 | 0.17 | 0.17 | 0.19 |
| 3 | 0.21 | 0.22 | 0.20 |
| 4 | 0.16 | 0.19 | 0.21 |
| 5 | 0.22 | 0.20 | 0.25 |
| 6 | 0.17 | 0.18 | 0.19 |
| 7 | 0.24 | 0.23 | 0.23 |
| 8 | 0.16 | 0.16 | 0.17 |
| 9 | 0.13 | 0.15 | 0.19 |
| 10 | 0.17 | 0.17 | 0.20 |
| Mean ± SD | 0.18 ± 0.03 | 0.18 ± 0.03 | 0.20 ± 0.02 |

**Table S5. Step width (m) during normal shoes, zero torque, and optimized torque conditions.**

| Participant | Normal Shoes | Zero Torque | Optimized Torque |
| --- | --- | --- | --- |
| 1 | 62.1 | 61.3 | 63.2 |
| 2 | 59.9 | 59.7 | 60.3 |
| 3 | 63.8 | 63.6 | 63.6 |
| 4 | 61.3 | 60.9 | 61.9 |
| 5 | 60.4 | 60.1 | 60.0 |
| 6 | 58.9 | 58.1 | 58.4 |
| 7 | 62.1 | 61.9 | 62.1 |
| 8 | 63.2 | 63.7 | 63.1 |
| 9 | 61.2 | 60.3 | 60.8 |
| 10 | 58.9 | 58.4 | 57.6 |
| Mean ± SD | 61.2 ± 1.7 | 60.8 ± 1.9 | 61.1 ± 2.0 |

**Table S6. Stance duration (%) during normal shoes, zero torque, and optimized torque conditions.**
